# Relationship between social interaction and health of the floating elderly population in China: an analysis based on interaction type, mode and frequency

**DOI:** 10.1186/s12877-023-04386-z

**Published:** 2023-10-16

**Authors:** Yiqing Xing, Liang Zhang, Yuelu Zhang, Ruibo He

**Affiliations:** 1 School of Political Science and Public Administration, Wuhan University, 299 Bayi Road, Wuchang District, Wuhan City, Hubei Province China; 2 School of Grammar and Law, Huazhong Agricultural University, 1 Shizishan Street, Hongshan District, Wuhan City, Hubei Province China; 3 School of Finance and Public Administration, Hubei University of Economics, 8 Yangqiaohu Avenue, Jiangxia District, Wuhan City, Hubei Province China

**Keywords:** Floating elderly population, Social interaction, Health, China

## Abstract

**Background:**

Although the health of the floating elderly population is an important part of active and healthy ageing, it is neglected in current Chinese society. Based on the general consensus that social interaction can affect the health of the floating population, this study explored whether the interaction type, mode and frequency influenced the health of the floating elderly population in China and investigated the variability of these influential effects.

**Methods:**

This study used the China Migrant Population Dynamic Survey Data 2017 and selected 5239 floating elderly individuals over 60 years old. Self-rated health was used to assess the comprehensive health status of respondents. Social interaction was measured by the interaction type, mode and frequency. Descriptive statistical analysis was used to analyse the health and social interaction status. An ordinal probit model was used to estimate the influential effects and differences on health caused by social interaction. The 2SLS model was used to examine the mutual causality relationship between interaction frequency and health, and a robustness test was conducted.

**Results:**

A total of 44.6% interacted with local residents, 14.2% participated in interaction activities based on geographical relations, and only 4.3% and 7% participated in interactions based on business and interest, respectively. Interacting with natives improved individuals’ self-rated health by 18.5%; specifically, geographical interaction increased self-rated health by 40.9%, occupational interaction increased it by 25.2%, interest-based interaction increased it by 41.2%, and interaction frequency improved the self-rated health (*β* = 0.128). In addition, sex, education level, personal income, and floating into the eastern region had a positive effect on individuals’ health. However, age, spouse and hukou exerted a negative effect.

**Conclusions:**

This study demonstrated that interacting with local residents could improve the health of floating elderly population, and revealed that interest-based interaction and their frequency had a positive impact on health. The government should speed up the construction of the voluntary service system and encourage the floating elderly population to realize their personal value in social interaction. In addition, the reform of the hukou system should be further promoted, so as to remove institutional barriers to the social interaction.

**Supplementary Information:**

The online version contains supplementary material available at 10.1186/s12877-023-04386-z.

## Background

With the rapid development of urbanization and industrialization, the number of floating populations in China has risen rapidly, providing the momentum and motivation for the high-speed development of China’s economy and society [1]. Population movement patterns in China have become domesticated and scaled-up in recent years. Coupled with the increase in population ageing and old age, the size of the floating elderly population has increased rapidly. Floating elderly population refers to the population over 60 years old who have left their place of registration for six months or more and are not registered in their current place of residence [2]. According to the China Floating Population Development Report 2018, from 2000 to 2005, the number of floating elderly individuals in China increased from 5.03 million to 13.04 million [3]. Compared with European and American countries [4], intergenerational relationships in traditional families are highly regarded in China, it is still a common phenomenon in current Chinese society for people to follow their children’s mobility to take care of their descendants [5], presenting typical Chinese characteristics. Therefore, the floating elderly population in China will increase further [6] and become a group worthy of consideration in combination with the continuous promotion of new urbanization and sustained intergenerational support.

Previous studies of the floating population have mainly focused on health vulnerability, inequality, and related influencing factors [7, 8]. The results showed that the health of the floating population is affected not only by individual-level factors such as age, sex, marital status and race [9–11], but also by social factors such as socioeconomic status and cultural habits [12–14]. Especially since the late 1950s, the hukou system implemented in China has excluded migrants from the scope of basic public services, resulting in unmet health needs [15, 16]. In addition, floating-level factors such as the floating time, reason and scope have an impact on the health of the floating population [17, 18]. However, compared with the widely studied floating youth, the health of the floating elderly population is more specific. On the one hand, from a full-life cycle perspective, the elderly population lacks health knowledge [19, 20] and has a high rate of chronic disease but a low rate of seeking medical treatment [21], which means a higher health risk [22]. On the other hand, the floating elderly population leaving their hometowns for various reasons, which cuts off their original social networks [23], and their decline in adaptive ability [24] have a negative impact on their health [25, 26]. However, the current research on the floating population focuses on the floating youth, insufficient attention is given to the China floating elderly population.

Considering the particularity of the floating elderly population, social interaction is an effective measure to reduce their health cost [27]. Social interaction refers to interaction with others in a formal or informal environment to maintain social relations [28], which means that social interaction is the core of social participation and can effectively contribute to the realization of active ageing [29, 30]. Existing research provides evidence that social interaction is correlated with the health of elderly individuals or migrants. The social relationship established through interaction can effectively prevent the social isolation of the floating population [31], reduce their inadaptability to new environments, increase their sense of belonging [32], and decrease their potential health risks [33, 34]. Social interaction is generally measured by interaction type, frequency and scope [35, 36]. In terms of interaction type, interacting with different objects has different impacts on the integration and health of rural migrants [37]. The greater the diversity of social interaction networks is, the more frequent the social interaction and the higher the older adult’s health expectation level [38]. However, studies on the influences of social interaction on the health of the floating elderly mainly focus on whether they interact or not, lacking a diverse perspective.

This paper focuses on the Chinese floating elderly population, and the research objectives include three aspects: (1) reveal the influence of social interaction on the health of the floating elderly population; (2) explore the most efficient type, frequency, and mode of social interaction that can potentially promote floating elderly people’s health; (3) through addressing the possible endogenous problems of this study, reveal the net effect of these influences. This research not only provides new directions for improving the health of the floating elderly population in China, but also provides scientific evidence to implement the “Healthy China” initiative and promote active ageing in China. Meanwhile, it also offers beneficial information related to the health of the floating elderly population for other Asian countries with family cultures similar to China, providing a basis for formulating forwards-looking policies.

## Methods

The data used in this article are derived from the 2017 China Migrants Dynamic Survey (CMDS) conducted by the Chinese Migrant Population Service Center. The survey was a cross-sectional survey covering 31 provinces, autonomous regions, and municipalities in China, using the probability proportionate to size sampling method and taking the floating population aged 15 years and older as the nationally representative subject of inquiry. The data mainly included five modules: family members, income and expenditure; health and public services; mobility and residence intention; employment; and social integration. The survey encompassed a wide range and the data were objective and reliable, with strong representativeness and comparatively good reliability and validity [39, 40].

A total of 169,989 samples were included in the data of the 2017 CMDS, and the floating elderly population over 60 years old served as the research subject of this paper. After removing 164,003 samples under 60 years old, and 747 samples with missing values for each variable and “do not know”, “unclear” and “unable to answer” responses, a total of 5239 valid samples were obtained, of which 3034 were male and 2025 were female.

### Variable

#### Dependent variable

For the purpose of this study, health was measured through Self-rated health. Self-rated health was measured by the question, “How do you feel about your own health status now?” The responses included four options: (1) unable to take care of yourself; (2) unhealthy but able to take care of yourself; (3) basically healthy; (4) healthy. To ensure the accuracy of the data, self-rated health was not simplified into dichotomous variables of only “good” and “bad” but retained the four categorical variables in the original questionnaire.

Objective illness status was used as a surrogate variable for self-rated health by asking the question, “Have you had any illness (injury) or physical discomfort in the last year?” The response included three options: (1) yes, the latest occurrence was within two weeks; (2) yes, the latest occurrence was more than two weeks ago; (3) “yes” to the first two items was coded as 1 and “no” as 0.

#### Independent variable

Social interaction was the independent variable in this study. Based on the theory of social capital, social interaction was measured with three dimensions: interaction type, interaction mode and interaction frequency [35–37].

**Interaction type** Interaction type was measured by the question, “Who do you locally interact with most in your spare time?” The responses included no interaction, other local residents, fellow villagers (whose hukou was moved to areas other than the local place and hometown), fellow villagers (whose hukou remained in the hometown), and fellow villagers (whose hukou was moved to the local area). Referring to Yang Juhua’s (2015) definition and measurement of interaction types [41], those who interacted with “fellow villagers (whose hukou was moved to the local area) or other local residents” were viewed as “interacting with local residents” and coded as 1, and other options were viewed as “interacting with nonlocal residents” and coded as 0.

**Interaction mode** Current research has classified social interactions into different types based on blood ties, geography, occupation and interests [42]. Modern social interactions are mainly built on occupational and interest relationships [43, 44], it is more important for floating elderly to interact with familiar residents from their place of origin instead of blood [45]. Therefore, this paper divides interaction types into three modes: geographical interaction, occupational interaction and interest-based interaction. The measurement question was, “Have you participated in any of the following activities in your local area since 2016?”, with the options of labour union, volunteer association, alumni association, hometown association, hometown chamber of commerce and others. Participation in activities such as “hometown association, alumni association, hometown chamber of commerce” were regarded as geographical interactions, participation in labour union activities was regarded as occupational interactions, and participation in volunteer association activities was regarded as interest-based interactions.

**Interaction frequency** Interaction frequency was measured by the question, “Have you ever taken an active part in volunteer activities since 2016?”. The options included (1) never; (2) occasionally; (3) sometimes; (4) often, reflecting the increasing frequency of interaction among the floating elderly population.

#### Concomitant variable

Some influences of demographic and socioeconomic factors were controlled and analysed, including sex, age, education level, marital status, hukou, personal income and insurance coverage of the floating elderly population. Hukou refers to the nature of individual household registration. It was measured through the question “your place of residence”, the option to answer the agricultural household registration was coded as 1, and the option to answer the urban household registration (same as non-agricultural household registration) was coded as 0. To make the data meet the normal distribution, individual income was processed by adding 1 and taking the logarithm. In addition, because the study subject was the floating elderly population, the factors of their mobility characteristics were controlled, including the mobility reason, mobility range and mobility time, among which the mobility time was measured by “current mobility time” in the questionnaire. The answer to this question was the mobility year and month, which was a numerical variable. To facilitate the research, mobility time was transformed into a continuous variable. May 2017 was taken as the time limit, and the respondents’ mobility year was subtracted from 2017. If the mobility month was less than 5, the mobility time was consistent with the mobility time calculated according to the year. If the mobility month was more than 4, the mobility year was increased by 1 based on the mobility time calculated according to the year.

#### Instrumental variable

Based on available studies, a reciprocal causation relationship may exist between interaction frequency and health status, which means that health status affects the interaction frequency with others and interaction frequency affects the health level of individuals [38]. Therefore, this paper used the instrumental variable method to solve the endogeneity problem of interaction frequency. Previous studies suggest that choosing the two variables of the degree of hometown culture maintenance and housing property as instrumental variables is appropriate for two reasons [46–48]. First, the degree of maintenance of hometown culture affects social interaction. The greater the degree of maintenance for floating elderly, the more difficult it is for them to integrate into the life of their new inflow region; therefore, they are more likely to isolate themselves and reduce participation in voluntary activities held there. Meanwhile, empirical research results show that the degree of hometown culture maintenance does not affect the health of floating elderly population [49, 50]. Second, the housing property affects social interaction. Compared with the floating population living in dormitories provided by employers or rental housing, self-owned housing can significantly improve the interaction between the floating population and local residents.

Drawing on relevant research [51], the degree of hometown culture maintenance was measured by the question “Do you agree with the statement that it is important for me to act in accordance with the customs of my hometown?” The question had four options: “completely disagree, disagree, basically agree, completely agree.” According to the degree of agreement of the interviewee, the variables were assigned a score from 1 to 4, with higher scores indicating a greater degree of cultural maintenance. Housing property was measured by the question, “Which of the following properties does your present house belong to?” The options included (1) unit/employer room; (2) rented private house-full rent; (3) rented private house-shared house; (4) public rental housing provided by the government; (5) self-purchased commercial housing; (6) self-purchased affordable housing; (7) self-purchased housing with small property rights; (8) borrowed housing; (9) place of employment; (10) self-built house and other informal places. According to the property rights of housing, in this paper, self-purchased housing was treated as “owning housing property rights” and coded as 1; rental housing, borrowed housing and place of employment were treated as “no property” and coded as 0. Appendix 1 shows the design and definition of the variables selected in this study.

### Statistical analysis

#### Descriptive statistical analysis

Descriptive statistical analysis was used to describe the health status, social interaction status and other covariates of the floating elderly population from the overall sample and the male and female subsamples.

#### Regression analysis

In the regression analysis, as the explained variable of self-rated health is an ordered categorical variable, the numbers 1–4 represent “very poor, poor, good, very good”, respectively. The ordinal probit model was used to test the effect of social interaction on the self-rated health of the floating elderly. The empirical model is as follows.

1 $${\text{Y}}_{\text{i}}={{\alpha }}_{0}+{{\beta }}_{1}{\text{S}}_{\text{i}}+{\lambda }\text{m}\text{i}+{\epsilon }\text{i}$$

where ${\text{Y}}_{\text{i}}$ refers to the self-rated health status of the floating elderly population; ${\text{S}}_{\text{i}}$ represents the social interaction status of the first i floating elderly population, including interaction type, interaction mode and interaction frequency; $\text{m}\text{i}$ is the matrix of control variables, including demographic characteristic variables, socioeconomic characteristic variables, and flow characteristic variables; ${\lambda }$ is the matrix of regression coefficients; ${\epsilon }\text{i}$ is the random error term; and the regression coefficient ${{\beta }}_{1}$ reflects the influence of social interaction on the health status of the floating elderly population.

#### The two-stage OLS model

As previously described, the endogeneity problem of social interaction frequency will lead to bias in the estimation results of the benchmark model. This study selected the degree of hometown culture maintenance and housing property of the floating elderly population as instrumental variables and used the two-stage OLS model. The regression equation was set as follows:

2 $${\rm{Stage}}\,{\rm{I}}:\,{{\rm{E}}_{\rm{i}}}\, = \,{\gamma _1}{{\rm{Z}}_{\rm{i}}}\, + \,\delta {{\rm{X}}_{\rm{i}}}\, + \,{\omega _{\rm{i}}}$$

3 $${\rm{Stage}}\,{\rm{II:}}\,{{\rm{Y}}_{\rm{i}}}\, = \,{\beta _3}{{\rm{E}}_{\rm{i}}}\, + \,{\beta _4}{{\rm{S}}_{\rm{i}}}\, + \,{\beta _5}{{\rm{C}}_{\rm{i}}}\, + \,\theta {{\rm{X}}_{\rm{i}}}\, + \,{\varepsilon _{\rm{i}}}$$

In the stage I model, ${\text{Z}}_{\text{i}}$ is the instrumental variable, ${\text{X}}_{\text{i}}$ is the other control variables, ${{\omega }}_{\text{i}}$ is the random disturbance term, and ${{\gamma }}_{1}$ and ${\delta }$ are the regression coefficient estimates, which reflect the influence of instrumental variables and other control variables on the interaction frequency, respectively. In the stage II regression model, ${\text{E}}_{\text{i}}$ is the predicted value of the stage I regression results, ${\text{S}}_{\text{i}}$ is the interaction type, ${\text{C}}_{\text{i}}$ is the interaction mode, ${\text{X}}_{\text{i}}$ is the other control variables, ${{\epsilon }}_{\text{i}}$ is the random disturbance term, and ${{\beta }}_{3}$, ${{\beta }}_{4}$ and ${{\beta }}_{5}$ are the regression coefficient estimates, which reflect the effects of the predicted values of the stage I regression results and other explanatory variables on the self-rated health of the floating elderly population.

## Results

### Descriptive statistical analysis

#### Characteristics of the floating elderly population

The interviewed floating elderly individuals had a mean age of 65.91 years, of which 57.8% were males, and 56.9% had agricultural hukou. The education level of the interviewed floating elderly population was generally low; 48.2% had an educational level of primary school or under, 29.9% had an educational level of junior middle school, and 21.9% had an educational level of high school and above. Men had a higher education level than women. The personal income of the interviewed floating elderly population was low; after being logged, the average personal income of the floating elderly population was 8.325, among which the average personal income was slightly higher for men than for women. In terms of participation in social security, 66.2% of the floating elderly population were enrolled in urban and rural resident medical insurance, and the rate of insurance participation for women (67.2%) was slightly higher than that for men. A total of 40.2% of the floating elderly population flowed to take care of their own elderly people, children and descendants; 34.8% flowed for work or business; 13.6% flowed for old-age care in other places; and 11.2% flowed for other reasons, including study and training, demolition and relocation, marriage and army. Among them, 45.4% of men flowed for work or business, while this percentage for women was only 20.1%. A total of 54.9% of women flowed to take care of their children and grandchildren, while only 24.8% of men flowed with their families. A total of 44.2% of elderly people flowed across provinces, 34.6% flowed across cities within provinces, and 21.2% flowed across counties within cities. There was an insignificant difference in the mobility range between male and female floating elderly adults. In terms of mobility time, the mobility time of the floating elderly population as a whole was 9.57 years (Table 1).

**Table 1:** Basic description of the floating elderly population

Variable	Total samples		Male floating elderly		Female floating elderly	
	Mean	SD	Mean	SD	Mean	SD
**Individual characteristic variable**						
Sex (female = 0)	0.577	0.494	1	0	0	0
Age	65.91	5.512	66.04	5.657	65.74	5.304
Hukou (non-agricultural = 0)	0.564	0.496	0.573	0.495	0.551	0.497
Marital status (without spouse = 0)	0.842	0.365	0.900	0.300	0.763	0.425
Primary education and below (other = 0)	0.479	0.500	0.406	0.491	0.579	0.494
Junior high school education (other = 0)	0.301	0.459	0.349	0.477	0.235	0.424
High school education and above (other = 0)	0.220	0.415	0.245	0.430	0.187	0.390
**Socioeconomic characteristic variable**						
Personal income	8.325	0.910	8.329	0.872	8.320	0.960
Insured status (not attend = 0)	0.654	0.476	0.641	0.480	0.672	0.469
**Flow characteristic variable**						
Mobility range						
Interprovincial mobility(other = 0)	0.442	0.497	0.442	0.497	0.442	0.497
Intercity mobility(other = 0)	0.346	0.476	0.345	0.476	0.347	0.476
Intercounty mobility(other = 0)	0.212	0.409	0.212	0.409	0.211	0.408
Mobility reason						
Working or doing business(other = 0)	0.346	0.476	0.452	0.498	0.202	0.401
Caring for children(other = 0)	0.406	0.491	0.301	0.459	0.549	0.498
Flowing for aged-care(other = 0)	0.136	0.343	0.141	0.348	0.130	0.337
Other flow reason	0.112	0.315	0.106	0.308	0.119	0.324
Mobility time	10.02	8.120	10.20	8.465	9.764	7.619
In-flows region						
Eastern region	0.405	0.491	0.397	0.489	0.416	0.493
Middle region	0.290	0.454	0.285	0.452	0.298	0.457
Western region	0.305	0.460	0.318	0.466	0.286	0.452
Sample size	5239		3034		2205	

#### Health status of the floating elderly population

**Fig. 1:**
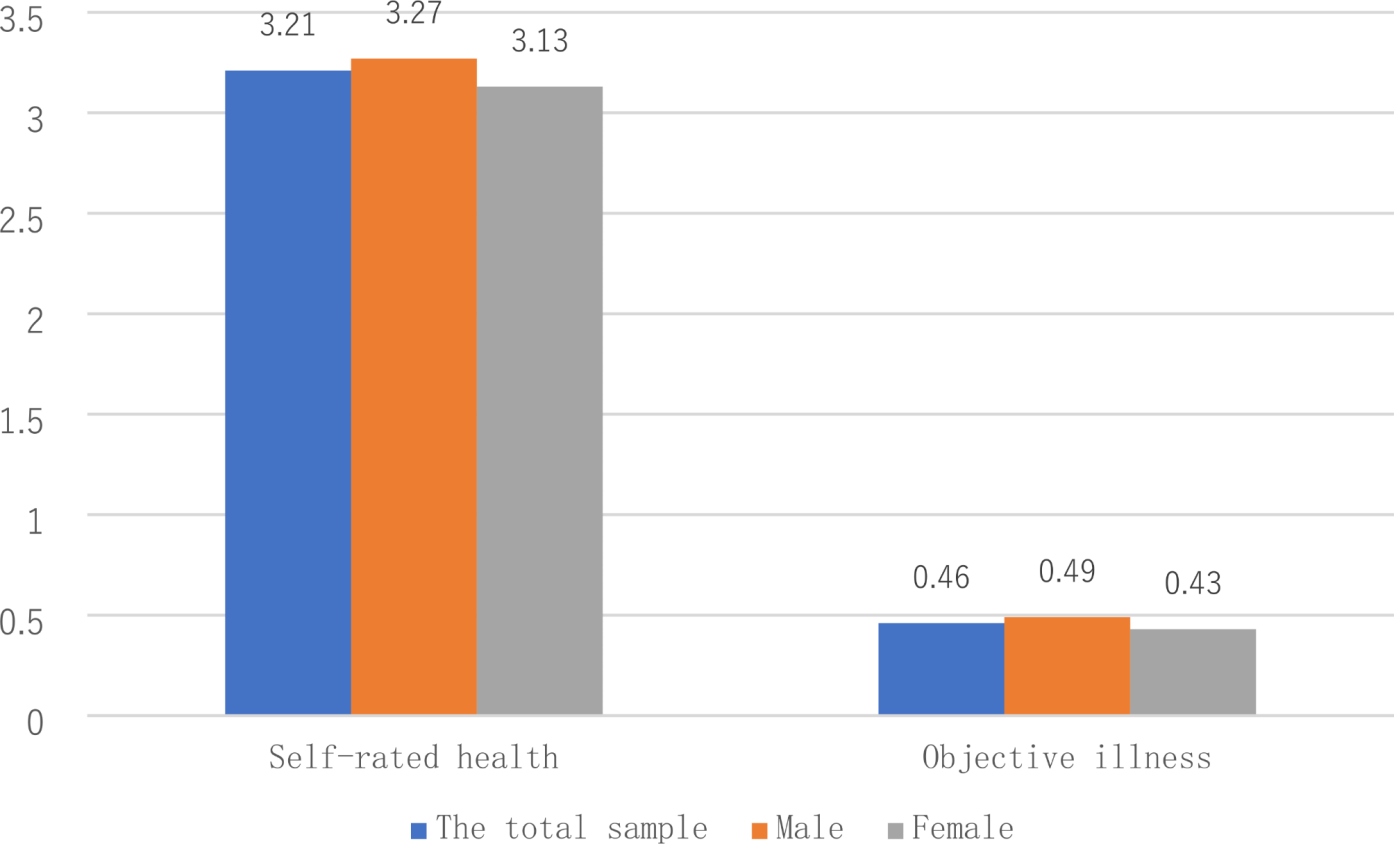
Health status of floating elderly individuals

On the whole, the mean self-rated health score of the interviewed floating elderly population was 3.21, indicating a good self-rated health status. As shown in Fig. 1, the mean self-rated health score for male and female floating elderly individuals was 3.27 and 3.13, respectively, with male scoring 0.14 points higher than female. Furthermore, 46% of the floating elderly population did not suffer from minor diseases in the last year, while 54% did. Among them, 57% of women and 51% of men had minor illnesses in the last year. The incidence of minor diseases in female floating elderly individuals was higher than that in male floating elderly individuals.

#### Social interaction status of the floating elderly population

**Fig. 2:**
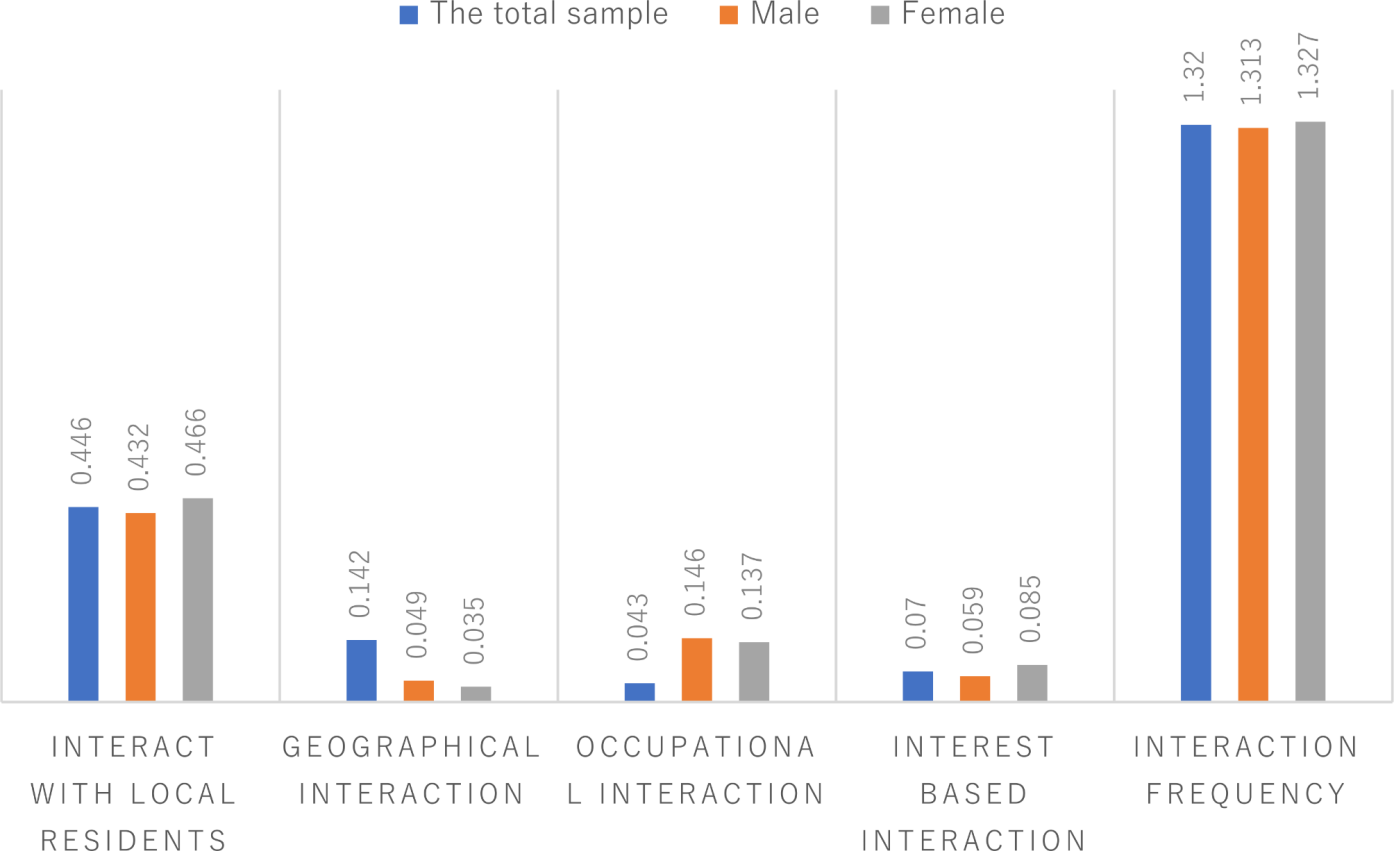
Social interaction status of the floating elderly population

Figure 2 reveals the social interaction of the floating elderly population in the local area. In terms of interaction type, 44.6% of the floating elderly population interacted with local residents, among which 46.6% were women and 43.2% were men. Regarding the interaction mode, 14.2% of the floating elderly population participated in interaction activities based on geographical relations, while only 4.3% and 7% were based on business and interest, respectively. Among them, 4.9% of men and 3.5% of women interacted based on geographical relations; 14.6% of men and 13.7% of women interacted based on business; and 5.9% of men and 8.5% of women interacted based on interest. Finally, for interaction frequency, the average interaction frequency of the floating elderly population was only 1.32.

### Regression analysis

#### Effect of interaction type on the health of floating elderly individuals

The stepwise regression method of Model 1–2 can clearly identify the influential effect of interaction type by enhancing the robustness of the regression results (Table 2). Interacting with local residents had a positive impact on the self-rated health of the floating elderly, with improved self-rated health by 18.5% compared with those who did not interact with local residents. With the gradual inclusion of control variables, the influential effects increased to 21.8%. Models 3–4 indicate that different interaction types had different effects on the self-rated health of the floating elderly population. Participating in interactions based on geographical relationship networks such as hometown associations and alumni associations improved the self-rated health of the floating elderly population by 40.9%, participating in interactions based on occupational relationship networks such as labour unions improved their self-rated health by 25.2%, and participating in interactions based on interest relationship networks such as volunteer interactions improved their health by 41.2%.

**Table 2:** Effects of social interaction on floating elderly

	M1	M2	M3	M4	M5	M6
Interaction type	0.185^***^	0.218^***^				
	(0.054)	(0.054)				
Geographical interaction			0.391^***^	0.385^***^		
			(0.080)	(0.081)		
Occupational interaction			0.285^**^	0.299^**^		
			(0.141)	(0.142)		
Interest based interaction			0.342^***^	0.357^***^		
			(0.113)	(0.113)		
Interaction frequency					0.099^***^	0.103^***^
					(0.038)	(0.038)
Sex	0.432^***^	0.386^***^	0.440^***^	0.393^***^	0.430^***^	0.383^***^
	(0.055)	(0.056)	(0.056)	(0.056)	(0.055)	(0.056)
Age	-0.073^***^	-0.065^***^	-0.071^***^	-0.063^***^	-0.072^***^	-0.064^***^
	(0.005)	(0.005)	(0.005)	(0.005)	(0.005)	(0.005)
Hukou	-0.053	-0.137^*^	-0.038	-0.121^*^	-0.063	-0.145^**^
	(0.071)	(0.073)	(0.072)	(0.073)	(0.071)	(0.073)
Marital status	-0.201^***^	-0.195^**^	-0.209^***^	-0.203^***^	-0.198^***^	-0.191^**^
	(0.075)	(0.076)	(0.076)	(0.076)	(0.075)	(0.076)
Education level	0.123^***^	0.130^***^	0.086^**^	0.094^**^	0.113^***^	0.120^***^
	(0.040)	(0.040)	(0.041)	(0.041)	(0.040)	(0.040)
Personal income	0.336^***^	0.305^***^	0.317^***^	0.286^***^	0.322^***^	0.292^***^
	(0.033)	(0.034)	(0.033)	(0.034)	(0.033)	(0.034)
Insured status	0.052	0.015	0.064	0.028	0.050	0.014
	(0.070)	(0.070)	(0.070)	(0.071)	(0.070)	(0.070)
Mobility range		-0.022		-0.016		-0.009
		(0.039)		(0.039)		(0.039)
Mobility reason		-0.201^***^		-0.195^***^		-0.193^***^
		(0.030)		(0.030)		(0.030)
Mobility time		-0.009^***^		-0.008^**^		-0.008^**^
		(0.003)		(0.003)		(0.003)
Eastern region		0.226^***^		0.239^***^		0.209^***^
		(0.068)		(0.068)		(0.068)
Middle region		-0.220^***^		-0.187^***^		-0.211^***^
		(0.070)		(0.070)		(0.070)
R^2^	0.050	0.054	0.054	0.058	0.050	0.057
Sample size	5239	5239	5239	5239	5239	5239

**Notes**: *、**、*** respectively denote the significance of 10%, 5% and 1%, Standard errors are contained within parentheses

From the above investigation, interactions based on interest had a greater impact on the health of the floating elderly population. However, in the context of active ageing, interaction activities based on interest have more practical significance for improving the health of the floating elderly population than traditional geographical interaction and occupational interaction. Thus, the influential effects of interest-based interaction frequency on the health of the floating elderly population were taken as the research priorities. The results of Models 5–6 indicate that the frequency of interest-based interaction had a significant positive effect on the self-rated health of the floating elderly. As the interest-based interaction frequency of the floating elderly population increased by 1 unit, their self-rated health increased by 10.3%. With the inclusion of more control variables, this influential effect decreased to a marginal extent but still had a significant positive effect at the 1% level on the whole.

Furthermore, as shown in Model 2, sex played a significant role in promoting the self-rated health of floating elderly people. Compared with floating elderly women, the self-rated health of floating elderly men improved by 38.6%. Age had a negative effect on the self-rated health of floating elderly individuals; as age increased by 1 unit, the probability of good self-rated health decreased by 6.5%. Compared with floating elderly individuals without spouses, the self-rated health of floating elderly individuals with spouses decreased by 19.5%. As personal income increased by 1 unit, the self-rated health of floating elderly individuals increased by 30.5%. As the mobility time increased by 1 year, the self-rated health of the floating elderly population decreased by 0.9%. Compared with elderly individuals who flowed into the western region, the probability of good self-rated health of elderly who flowed into eastern region increased by 22.6%, and who flowed into middle region decreased by 22.6%.

### Robustness test

#### Endogenous test

There may exist an endogenous relationship between the interaction frequency and the health of floating elderly. Instrumental variable method was used to solve the endogenous problem of interaction frequency. As previously described, the degree of hometown culture maintenance and housing property were selected as instrumental variables. Table 3 reports the regression results of the instrumental variables. First, the stage I regression results report the influence of various variables on interaction frequency. Both the degree of hometown culture maintenance and housing property had significant effects on the interaction frequency of the floating elderly. The degree of hometown culture maintenance had a negative impact on the interaction frequency of the floating elderly. As the degree of hometown culture maintenance increases, the frequency of interaction among floating elderly decreases. Compared with the floating elderly population without property rights, such as renting or borrowing housing, the interaction frequency among floating elderly people with property rights, such as buying or building houses, increased by 7.4%. Second, the stage II regression results report the influential effects of each variable on the self-rated health of the floating elderly population after adding control variables. As an endogenous explanatory variable, interaction frequency still had a positive effect on the self-rated health of the floating elderly population after the inclusion of instrumental variables with a regression coefficient of 0.668, which was significantly higher than the basic regression results (0.103). After the treatment of the endogeneity of interest-based interaction frequency, its influential effect on the self-rated health of the floating elderly population not only had robustness but also increased further. Among the other variables, interaction type and interaction mode had a positive impact on the self-rated health of the floating elderly population at a significance level of 1%, which was consistent with the influential effect of the benchmark regression results.

**Table 3:** Instrumental variable estimation results

variable	stage I	stage II
Interaction frequency		0.070^***^
		(0.021)
the degree of hometown culture maintenance	-0.039^***^	
	(0.011)	
Housing property	0.117^***^	
	(0.020)	
Region characteristics	controlled	
P(DWH)	0.014	
F (weak instrumental variable test)	20.51	
P (overidentification test)	0.297	
R^2^	0.239	
Sample size	5239	

**Notes**: *、**、*** respectively denote the significance of 10%, 5% and 1%, Standard errors are contained within parentheses

Instrumental variables need to meet three test conditions. First, we test for the endogeneity of the explanatory variables. The DWH test was used in this paper, which is a heteroscedastic robust test method. The P value of 0.014 was obtained, which is less than 0.05, so interaction frequency can be considered an endogenous explanatory variable. Second, there is a correlation between instrumental variables and endogenous variables. It can be understood that there was no problem of weak instrumental variables in this paper since the F-statistic value in stage I was greater than 10. The third is the exogeneity of the instrumental variables. Since the P value of the overidentification test was 0.297, which exceeded the critical value of 0.1, the null hypothesis that “the degree of hometown culture maintenance and housing property are exogenous and have no correlation with the disturbance term” was accepted. The satisfaction of the three conditions above indicates that it is reasonable to choose the degree of hometown culture maintenance and housing property as instrumental variables to determine the endogeneity problem of this paper.

#### Surrogate variable test of the explained variable

As the surrogate variable of the explained variable self-rated health, illness status examines the effect of social interaction on the objective physical health of the floating elderly population. To be clear, because objective disease status and self-rated health status are two variables with opposite assignment directions and social interaction had a positive effect on the self-rated health of the floating elderly population, only when social interaction had a negative effect on objective disease for the floating elderly population would the results be robust.

Social interaction had a negative effect on the objective illness status of the floating elderly population (Table 4). Interacting with local residents reduced the probability of the floating elderly population having minor ailments by 3.6%. Among the interaction modes, interaction through different organizations, such as geography, business and interest relationships, reduced the probability of the floating elderly population having minor ailments by 46.4%, 18.6% and 7.9%, respectively. In addition, there was a negative correlation between the interest-based interaction frequency and the objective illness status of the floating elderly population; with more frequent interaction frequency, the probability of the floating elderly population having minor ailments in the past year decreased by 12.2%, indicating that social interaction had a robust effect on the health of the floating elderly population.

**Table 4:** Estimation results of social interaction on the objective health of floating elderly individuals

Substitute variable	M14	M15	M16	M17
Interaction type	-0.036^**^			-0.025^**^
	(0.016)			(0.012)
Geographical interaction		-0.464^***^		-0.476^***^
		(0.147)		(0.148)
Occupational interaction		-0.186^**^		-0.168^**^
		(0.084)		(0.085)
Interest based interaction		-0.079^**^		-0.081^**^
		(0.039)		(0.041)
Interaction frequency			-0.122^***^	-0.141^***^
			(0.041)	(0.045)
Control variable	controlled	controlled	controlled	controlled
R^2^	0.015	0.017	0.017	0.019
Sample size	5239	5239	5239	5239

Notes: *、**、*** respectively denote the significance of 10%, 5% and 1%, Standard errors are contained within parentheses

### Heterogeneity analysis of explanatory variables with different dimensions

As previously described, the interaction type had a positive effect on the health of the floating elderly population. To further analyse the influential effects of different interaction types on the self-rated health of the floating elderly population, a broader classification of interaction types was adopted to provide insight into the difference in interaction types on the health of the floating elderly population. Among them, interactions with local residents include two categories: fellow villagers (whose hukou was moved to the local area) and other local residents. Non-local residents include three categories: fellow villagers (whose hukou remained in the hometown), fellow villagers (whose hukou was moved to the areas other than local place and hometown), and other outsiders. As presented in Table 5, in the local interaction, interacting with other local residents had a positive effect on the self-rated health of the floating elderly population with an influence coefficient of 0.341, which matched the result mentioned above. In interactions with fellow villagers with different hukou, the interaction only with fellow villagers whose hukou remained in their hometown had a positive impact on the self-rated health of the floating elderly population, with a coefficient of 0.285 and a significant correlation at the level of 1%. Additionally, the coefficient of the relationship between interaction with local residents whose hukou moved to the local area and self-rated health among floating elderly population was negative (-0.021), although it was not statistically significant.

**Table 5:** The estimation results of multiple interaction types on the health of floating elderly people

Interaction type	M18
Fellow villagers (whose hukou was moved to the local area).	-0.021
	(0.147)
Fellow villagers (whose hukou remained in the hometown)	0.285^***^
	(0.093)
Fellow villagers (whose hukou was moved to the areas other than local place and hometown)	0.098
	(0.221)
Other local residents	0.341^***^
	(0.061)
Other outsiders	0.351^***^
	(0.115)
R^2^	0.056
Sample size	5239

Notes: *、**、*** respectively denote the significance of 10%, 5% and 1%, Standard errors are contained within parentheses

## Discussion

This study focused on the floating elderly population, which is often overlooked. Based on the double adversity characteristics of “mobility” and “ageing”, this study is the first to estimate the impact of diversified social interactions on the health of the floating elderly population from the perspective of the type, frequency and mode of social interaction. The results show that the self-rated health of the floating elderly population was good, while their objective illness status was poor. Existing studies have confirmed that the floating elderly population has weak health knowledge and a low proportion of health records [19, 52], which leads them to be so optimistic about their own health expectations that they ignore their actual health risks. This finding suggests that we should pay more attention to the provision of public health services for the floating elderly population, increase health education for this population, and improve their health literacy.

The interaction type was conducive to the health of the floating elderly population. Interacting with local residents significantly improved their self-rated health status. This finding breaks through the current research that only discussed the impact of interaction and non-interaction on the health of the floating elderly [38], and further discovers the importance of interaction with the local people. This is related to China’s traditional Confucian culture, which values interpersonal relationships and emphasizes “collectivism” [53]. Chinese interpersonal interaction follows the principle of particularism based on relationships and human feelings, and individuals hope to be recognition by people around [54]. This phenomenon is more prominent in the floating elderly, who are more eager to enter the local circle and get the recognition and acceptance of local residents [55]. When interacting with local residents, they take local norms as their criteria of action and generate the identity of “native”, which greatly improves their sense of belonging and adaptability in an unfamiliar city [56], and thus produces better self-rated health.

After subdividing the interaction types, we found that “hukou location” was a very important factor in the process of interacting with fellow villagers: interaction between the floating elderly population and residents whose hukou was in their hometown improved self-rated health, while interaction with residents whose hukou was moved to the local area reduced self-rated health. A possible reason for this phenomenon is that the floating elderly population compare themselves with fellow villagers in the process of interacting with them. What is bound with the local household registration is the accessibility and availability of public services such as medical care and elderly care [57]. When they find that fellow villagers whose hukou was moved to the local area have a “comparative advantage” in enjoying local public services and medical services, their local life satisfaction and self-rated health decline. This is basically consistent with existing studies [7, 16] and reflects the negative effects triggered by the reform of China’s hukou system. The Chinese government introduced a reform design for the hukou system in 2014, abolishing the distinction between agricultural and non-agricultural hukou. However, the study results indicate that China still needs to further advance the reform of the hukou system, truly break through the gap between residents with urban and rural hukou in their enjoyment of basic local public services, and encourage the floating population with different hukou to enjoy various public services fairly.

Interaction modes had different effects on the health of the floating elderly population. Floating elderly individuals interacted by participating in different modes of activities; the more varied the modes were, the better their self-rated health. Interaction based on interest had the greatest impact on health, and interaction based on geographical advantage and occupational relationship had the second greatest impact. Fei Xiaotong’s “Pattern of Difference Sequence” holds that people interact mainly in geographical relations, which is a typical phenomenon of interpersonal relations in Chinese society [58]. Chinese traditional culture “Confucianism” also advocates “family culture”, family and clan become the main social community [59]. Social interaction depends on blood relationship, and the whole society is also an acquaintance society [60]. The research results of this paper break through the traditional viewpoints of Chinese sociologists [61, 62], highlight the important status of interest-based interaction, which is more consistent with China’s situation in the context of the new era, and provide an empirical basis for the community of inflow regions to actively construct a voluntary service system for elderly individuals. Furthermore, the reasons for this phenomenon are worth exploring. The possible reasons are as follows. First, with the improvement of material living standards, the hierarchy of needs of elderly individuals has been enhanced, and they are increasingly pursuing the satisfaction of spiritual life. Previous studies have shown that interactions based on interest are more colourful and diverse and can better address the needs of elderly individuals, increasing their sense of belonging and thereby improving their health [63]. Second, based on social capital theory, the interaction relationship is related to scale [64]. Due to its nature, interest-based interaction tends to have a larger scale and greater internal differences than geographical interaction, which is conducive to the greater influential effect of interest-based interaction.

Interaction frequency played a positive role in the health of the floating elderly population. The greater the frequency of interest-based interaction is, the better the self-rated health of the floating elderly population. Interaction based on interest relationships is a kind of community social capital investment that can transform the trust and cooperation resources to support collective action, which is consistent with existing research [65]. With the changes of China’s social structure, the floating elderly need more heterogeneous resources from a broader social network [66]. A conscious social network engagement based on interests, hobbies, and values can help them communicate deeply [67]. The floating elderly population with frequent interaction in the local area can form a stronger sense of identity, which can enhance their emotional satisfaction and health expectations [68, 69]. This finding also validates Putnam’s explanation of the relationship between social capital and well-being outcomes [70], and provides empirical evidence for the community to increase the number of voluntary activities.

This paper verified the relationship between social interaction and the health of the floating elderly population, which is of practical significance to promote healthy ageing, active ageing and the equalization of public services for the floating population against the background of ageing and urbanization. First, the Chinese government should accelerate the construction of a community support system, emphasize the safeguarding role of the community, establish a paired relationship between local residents and the floating elderly population, and create opportunities for the active participation of floating elderly individuals. Furthermore, the establishment of a voluntary service system to realize the value of the floating elderly population in the context of active ageing should be explored, which will make voluntary social organizations become the main platform for floating elderly individuals to engage in social communication, encouraging the floating elderly population to realize their personal value in communication and interaction. In addition, it is necessary to actively promote and strengthen the reform of the hukou system and break down the institutional barriers of social interaction and health improvement for the floating elderly population.

There are three limitations to this study. First, due to data limitations, mental health indicators, BMI indicators, and cognitive ability were not included in the measurement of the health of floating elderly individuals. This needs to be improved and perfected in follow-up studies in the future. However, the focus of this paper is the influential effect of social interaction on the health of the floating elderly population, and self-rated health is an individual’s health evaluation according to illness status, living environment and life satisfaction and reflects the comprehensive health level of the floating elderly population to a certain extent [71], which has no impact on the purpose of the study. Second, social interaction has rich connotations and extensions, such as scale, depth, density, and homogeneity. Due to limitations of the study’s purpose and the data, not all interaction dimensions were included in the research scope, and only interaction type, mode and frequency were selected as measurement indicators, focusing on the measurement of interaction density and scale, which led to a lack of exploration of the impact of interaction depth. Third, this study focused on the causal relationship between social interaction and the health of the floating elderly population and did not examine the mechanism and path of action, which needs further in-depth research in the future.

## Conclusions

This study found that social interaction has a positive effect on the health of the floating elderly population. Specifically, interacting with local residents significantly improves the health of the floating elderly population, but the effect of interacting with fellow villagers on the health of the floating elderly population is moderated by the location of hukou. Interaction based on interest has the greatest impact on the health of the floating elderly population, and the interaction frequency is positively correlated with their health, highlighting the importance of interest-based interaction. This study provides an empirical basis for optimizing public health policies and promoting the equalization of public health services. Moreover, under the situation of the population ageing trend and China’s new-type urbanization transformation, the research findings can provide specific implementation paths for promoting the healthy ageing and active ageing of the floating elderly population in China.

### Electronic supplementary material

Below is the link to the electronic supplementary material.

**Supplementary Material 1: Appendix 1** Design and definition of variables

## Data Availability

This study used the de-identified data from the 2017 China Migrants Dynamic Survey (CMDS), which were collected by the Chinese Migrant Population Service Center. These data are not publicly available, and restrictions apply to the availability of these data. Users need to apply in the name of an institution. Questionnaires and the datasets are available upon reasonable request and with permission of Chinese Migrant Population Service Center. The datasets can be requested as: (a) visit to: https://www.chinaldrk.org.cn/wjw/#/data/classify/population/yearList; (b) select the 2017 CMDS data, click on download, and then you can see a login page, where you should register and login in the name of the institution; (c) after obtaining access, you can see the procedure as: Data Preliminary Review-Material Submission-Data Final Review-Data Release.
