# Supplementary material for: Relationship between social interaction and health of the floating elderly population in China: an analysis based on interaction type, mode and frequency
Source: BMC Geriatr. 2023 Oct 16;23:662. doi: 10.1186/s12877-023-04386-z (PMC10580520; doi:10.1186/s12877-023-04386-z)
Supplement: Supplementary file 1 — Supplementary Material 1: Appendix 1 Design and definition of variables [file 12877_2023_4386_MOESM1_ESM.docx]

**Appendix 1 Design and definition of variables**

| Variable | Definition | Maximum | Minimum |
| --- | --- | --- | --- |
| **Explained variable：health status** | | | |
| Self-rated health | 1=very poor, 2=poor, 3=good, 4=very good | 4 | 1 |
| Illness status | 1= ill within the last year,  0= no illness within the last year | 1 | 0 |
| **Explanatory variable：social interaction** | | | |
| 1.Interaction type ^a^ | 1= interact with local residents,  0= interact with non-local residents | 1 | 0 |
| 2.Interaction mode |  |  |  |
| Geographical interaction ^b^ | 1=yes, 0=no | 1 | 0 |
| Occupational interaction ^c^ | 1=yes, 0=no | 1 | 0 |
| Interest based interaction ^d^ | 1=yes, 0=no | 1 | 0 |
| 3.Interaction frequency | 1=never, 2=occasionally, 3=sometimes, 4=often | 4 | 1 |
| **Control Variables** | | | |
| 1.Individual characteristic variable |  |  |  |
| Sex | 1=male, 0=female | 1 | 0 |
| Age | Continuous variable | 96 | 60 |
| Hukou | 1=agriculture hukou, 0=non-agriculture hukou | 1 | 0 |
| Marital status | 1= with spouse, 0=without spouse | 1 | 0 |
| Education level | 1=primary education and below, 2= junior high school education, 3= high school education and above | 3 | 1 |
| 2.Socioeconomic characteristic variable |  |  |  |
| Personal income | Log of personal income | 11.70 | 4.38 |
| Insured status | 1=yes; 0=no | 1 | 0 |
| 3. Flow characteristic variable |  |  |  |
| Mobility range | 1= interprovincial mobility, 2=intercity mobility, 3=intercounty mobility | 3 | 1 |
| Mobility reason | 1= working or doing business, 2= caring for children, 3=flowing for aged-care，4=other | 4 | 1 |
| Mobility time | Continuous variable | 70 | 0 |
| In-flows region | 1= eastern region, 2= middle region, 3= western region | 3 | 1 |
| **Instrumental variable** | | | |
| Mobility mode | 1= flowing with others, 0=independent flow | 1 | 0 |
| Housing property | 1=have housing property，0=no housing property | 1 | 0 |

**Notes:** a represents who you interact with locally; b represents social interactions based on activities related to the geographical location of one's hometown; c represents interpersonal communications based on occupation or industry; d represents communications based on the same interests.
